# Lipoxins and Annexin-1: Resolution of Inflammation and Regulation of Phagocytosis of Apoptotic Cells

**DOI:** 10.1100/tsw.2006.259

**Published:** 2006-12-06

**Authors:** Michael Scannell, Paola Maderna

**Affiliations:** UCD School of Medicine and Medical Science, UCD Conway Institute of Biomolecular and Biomedical Research, University College Dublin, Belfield, Dublin 4, Ireland

**Keywords:** lipoxin, annexin-1, resolution of inflammation, phagocytosis, macrophages, neutrophils, apoptotic cells

## Abstract

Phagocytosis of apoptotic cells plays a pivotal role in developmental processes and in the resolution of inflammation. Failed or delayed clearance of apoptotic cells can result in chronic inflammation. Furthermore, clearance of apoptotic cells leads to release of anti-inflammatory cytokines. Recent evidence has shown that endogenous mediators can regulate such processes. In this article, we will review the recognition and signaling mechanisms involved in the phagocytosis of apoptotic cells as well as the role of endogenous compounds that play a relevant role in the modulation of inflammation. The first of these endogenous local mediators to be described are lipoxins (LXs). LXs and aspirin-triggered LXs (ATLs) are considered to act as “braking signals” in inflammation, limiting the entrance of leukocytes to the site of inflammation through inhibition of neutrophil and eosinophil trafficking. LXs are actively involved in resolution of inflammation, stimulating nonphlogistic phagocytosis of apoptotic cells by macrophages. LXA_4_ and ATLs elicit cellular responses by interacting with a G protein -coupled receptor (ALXR) that is expressed in various cell types. ALXR, originally identified as a low-affinity N-formyl-methionyl-leucyl-phenylalanine receptor-like 1, can bind pleiotropic ligands, i.e., both lipid and peptides, including the glucocorticoid-inducible protein, annexin-1. Interestingly, a role for annexin-1 in phagocytosis has recently emerged. Understanding the role and mechanism of the powerful anti-inflammatory and proresolution actions of endogenous compounds can be a useful tool in the development of potential therapeutics in resolving inflammatory diseases.

## INTRODUCTION

The resolution of inflammation is a dynamically regulated process that involves the suppression of proinflammatory gene expression and leukocyte migration, resulting in programmed leukocyte death by apoptosis followed by clearance by phagocytes[1,2,3]. The biosynthesis of specific mediators that actively promote the resolution of inflammation has been demonstrated in this context[4,5]. An initial acute response involves leukocyte recruitment and activation. During this initial phase, proinflammatory mediators including leukotriene (LT) $B_4$, the cysteinyl LTs, and prostaglandins (PGs) evoke potent chemotactic responses of leukocytes whose activation is coupled to the production of proinflammatory (Th1-derived) cytokines at sites of inflammation[6]. To facilitate resolution, a second phase of lipid mediators may be produced, favoring agents with "proresolution activities" including lipoxins (LXs) and the more recently described resolvins and protectins[3,4,5,6,7,8]. As a consequence, the recruitment of polymorphonuclear neutrophils (PMNs) is ended and they can undergo apoptosis. The subsequent removal of apoptotic cells is an important step in sparing tissue from exposure to the noxious and immunogenic contents of necrotic cells, and is a prerequisite to restore normal tissue function[9,10,11,12]. Clearance of apoptotic cells is mediated by professional phagocytes, such as macrophages (Mϕ) and immature dendritic cells, and by a variety of nonprofessional phagocytes that engulf apoptotic cells with less efficiency[13,14]. The engulfment of apoptotic cells is associated with the release of anti-inflammatory mediators, such as transforming growth factor-β1 (TGF-β1), interleukin (IL)-10, and $PGE_2$, and with inhibition of the secretion of proinflammatory mediators, such as tumor necrosis factor-α (TNF-α), as demonstrated by *in vitro* and *in vivo* studies[15,16,17]. Recently, it has been demonstrated that phagocytosis of apoptotic cells triggers secretion of vascular endothelial growth factor, which is critical for repair of endothelial and epithelial injury[18].

Defective clearance of apoptotic cells has been associated with inflammatory and autoimmune diseases[1,10,19,20]. Given that recognition and engulfment of apoptotic cells is an important process in the resolution of inflammation, a positive regulation of the capacity of Mϕ for phagocytosis of dying cells represents a potential therapeutic target in the control of inflammatory disease. Here we will review the recognition and signaling mechanisms involved in phagocytosis of apoptotic cells and will discuss the role of LXs and of LX receptor agonists in the modulation of resolution of inflammation

## RESOLUTION OF INFLAMMATION AND PHAGOCYTOSIS OF APOPTOTIC CELLS

### Apoptotic Cell-Phagocyte Interaction

The recognition and clearance of apoptotic cells by phagocytes involves multiple components on the surface of the apoptotic cells and on the phagocytes, apoptotic cell-associated ligands, as well as a variety of "bridging" molecules frequently derived from plasma constituents (Fig. 1). In order for the apoptotic cells to be recognized and ingested, membrane alterations must occur in a way that differentiates them from viable cells[9,10,11,12]. Among the multiple changes on the surface of the apoptotic cells that facilitate their recognition, the exposure of phosphatidylserine (PS) is a central player in the recognition and engulfment of apoptotic cells[21,22]. However, while necessary, PS exposure is not sufficient to complete clearance of apoptotic cells[23]. Interestingly, the engulfment process seems to require PS exposure not only on the apoptotic target, but also on phagocytes[24], even if at lower level, suggesting that other recognition factors might be expressed on apoptotic cells to facilitate their uptake. Recently, annexin-1 was found to colocalize with PS on apoptotic cells and was associated with efficient tethering and internalization[25]. Annexin-1 is exported from the cytosol to the plasma membrane of apoptotic cells by a mechanism dependent on caspase activation and is required for the clustering of overexpressed PS receptor around apoptotic cells[25]. Also, a defect in the clearance of apoptotic cells was observed in *Caenorhabditis elegans*, when the homologue gene of annexin-1 was silenced by small interfering RNA[25].

In addition to these changes on apoptotic cells, a negative regulation of phagocytosis might be used to prevent ingestion of viable cells mediated by molecules such as CD47, a surface marker recognized by signal regulatory protein[26,27], and CD31, a member of immunoglobulin superfamily that has been proposed to prevent phagocyte ingestion of viable cells by transmitting "detachment" signals[28]. Ligation of CD31 on viable leukocytes promotes their detachment from Mϕ, whereas such CD31-mediated detachment is disabled in apoptotic leukocytes, promoting tight binding and Mϕ ingestion of dying cells[28].

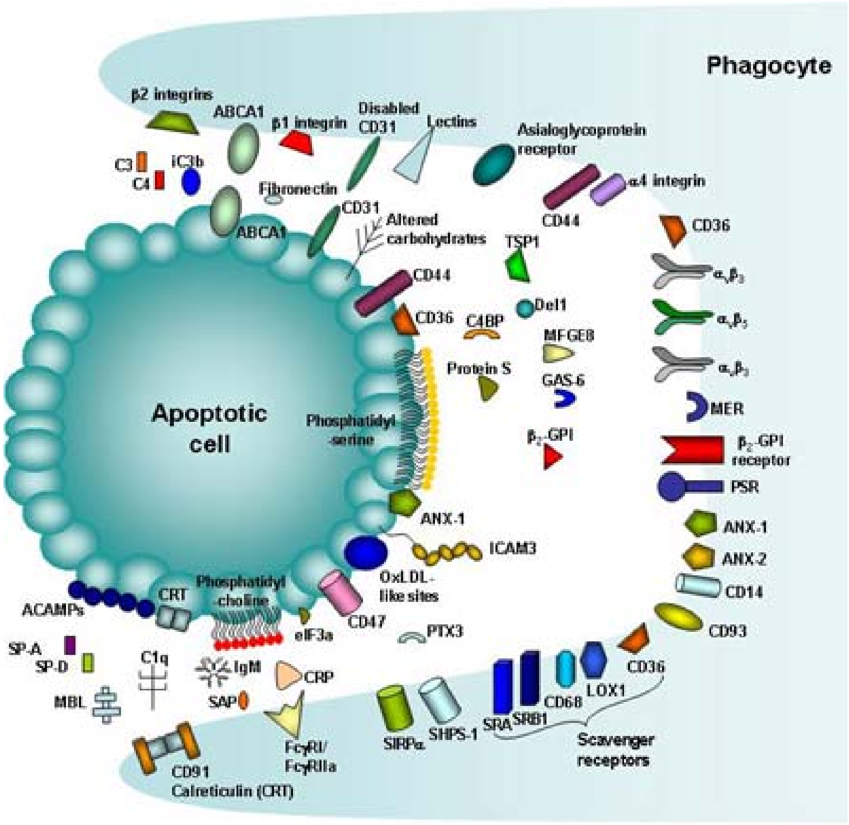

**FIGURE 1.** The myriad of molecules involved in the regulation of phagocytosis of apoptotic cells. Phagocytic receptors, bridging molecules, and apoptotic ligands interact to coordinate the phagocytosis of apoptotic bodies. These include: C3, complement protein C3; C4, complement protein C4; β2 integrin, CD11/CD18; iC3b, complement protein iC3b; ABCA1, ATP-binding cassette transporter 1; β1 integrin, CD29; CD31, platelet endothelial cell adhesion molecule-1 (PECAM1); CD44, receptor for hyaluronic acid; α4 integrin, CD49d; CD36, thrombospondin receptor; TSP1, thrombospondin 1; Del1, developmental endothelial locus 1; $\alpha_v\beta_3$ and $\alpha_v\beta_5$ integrins that bind vitronectin; MFGE8, milk fat globule-EGF factor 8; C4BP, C4-binding protein; GAS-6, growth arrest-specific 6; MER, MER tyrosine kinase; $\beta_2$-GPI, $\beta_2$ glycoprotein-1; $\beta_2$-GPI receptor, $\beta_2$ glycoprotein-1 receptor; PSR, phosphatidylserine receptor; ANX-1, annexin-1; ANX-2, annexin-2; CD14, lipopolysaccharide receptor; ICAM3, intercellular adhesion molecule 3; OxLDL-like sites, oxidized low-density lipoprotein-like sites; LOX1, lectin-like low-density lipoprotein receptor-1; CD68, macrosialin; SRB1, scavenger receptor class B1; SRA, scavenger receptor class A; CD47, integrin-associated protein (IAP); SHPS-1, SHP protein-tyrosine phosphatases substitute-1; SIRPα, signal regulatory protein α; eIF3a, eukaryotic translation initiation factor 3; PTX3, long pentraxin 3; CRP, C-reactive protein; FcγRI/FcγRIIA, Fc fragment of immunoglobulin G receptor I and IIA; SAP, serum amyloid P component; IgM, immunoglobulin M; CRT, calreticulin; C1q, complement protein C1q; ACAMPs, apoptotic cell-associated molecular patterns; SP-A, surfactant protein A; SP-D, surfactant protein D; MBL, mannose-binding lectin; CD91, α2 macroglobulin receptor.

Phagocytes, in turn, show significant redundancy in recognition strategies and are able to use many receptors at the same time, reflecting multiple phases in the interaction between apoptotic cells and phagocytes (Fig. 1). Some receptors may simply play a role in tethering of phagocyte to apoptotic cells without generating a signal, whereas others may activate a signal pathway leading to cytoskeleton rearrangements and engulfment[1,10,12]. Since the identification of the macrophage vitronectin receptor ($\alpha_v\beta_3$) as the first receptor involved in recognition and engulfment of apoptotic cells[29], numerous molecules involved in phagocytosis of apoptotic cells, belonging to many different receptor families, were characterized. These include PS receptor, the scavenger receptors, lectins, the receptor tyrosine kinase Mer, the lipopolysaccharide receptor CD14 (which binds to the Ig superfamily member adhesion molecule ICAM-3), and members of the collectin family and their receptors, CD91 and calreticulin (Fig. 1)[1,10,11,12]. Conflicting data have emerged from the first experimental evidence of the existence of a PS receptor[30], since it has been shown that the *psr* gene described originally does not encode a PS receptor, but a nuclear protein that plays a role in development and differentiation, probably as a regulatory protein related to the iron-oxidase family of proteins[31,32]. In addition, studies in PS receptor knockout mice have provided contradictory data regarding the role of the PS receptor in apoptotic cell clearance. In one study, mice deficient for the PS receptor presented lung developmental abnormalities and occasional brain hyperplasia, which were associated with increased numbers of nonphagocytosed apoptotic cells[33]. Another study showed that knockout of the *psr* gene in mice resulted in severe developmental defects and in a reduced number of M$\phi$ in fetal livers of knockout versus wild-type animals associated with impaired clearance of apoptotic cells[34]. However, in a third study, it was demonstrated that ablation of the PS receptor function in mice caused a delay in the development and differentiation of multiple organs during embryogenesis, but that the clearance of apoptotic cells was normal[35].

Some receptors interact directly with apoptotic cells, however, some interactions occur indirectly through bridging proteins, such as plasma-protein $\beta_2$-glycoprotein I[36], the product of growth arrest specific gene 6 (Gas6) that binds to the Mer kinase[37], the protein milk-fat globule epidermal growth factor 8 (MFG-E8) that bridges with $\alpha_v\beta_3$ integrin [38], and serum-derived protein S[39]. In addition, opsonization of apoptotic cells by components of the innate immune system, such as complement factors and other opsonins-like collectins, pentraxins, and anticoagulant proteins, facilitate and modulate the clearance of apoptotic cells by classical phagocytic receptors even if they seem to be involved particularly in later stages of the cell death process[40,41].

It is reasonable to predict that dying cells may release attraction signals to induce the migration of professional phagocytes to sites of apoptosis. Such signals may be chemotactic, phagocytic, or both. In this context, it is noteworthy that the phospholipid lysophosphatidylcholine, released from apoptotic cells in a caspase 3–dependent manner, has been identified as a “recruitment” signal being chemotactic for monocytes and M$\phi$[42].

## Signaling Pathways in Phagocytosis of Apoptotic Cells

Phagocytosis of apoptotic cells is mediated by numerous recognition ligands and their cognate receptors and by redundant, but probably interacting, signaling pathways. The intracellular pathways involved in phagocytosis of apoptotic cells were identified in the nematode *C. elegans*, which provides a powerful genetic system for the study of programmed cell death[43,44]. On the basis of their genetic interactions, the engulfment genes fall into two partially redundant pathways that possibly converge at a common effector, ced-10 (mammalian homolog Rac-1)[43]. In the first pathway, the proteins CED-2, CED-5, and CED-12 (mammalian homologues CrkII, Dock180, and ELMO, respectively) control cytoskeletal events required for the extension of phagocytic arms[44,45]. The second group includes CED-7 (ABCA1), the candidate receptor CED-1, and the adaptor protein CED-6[44,45]. Recently, Yu et al. identified and characterized another gene, *dyn-1*, involved in the phagocytosis of cell corpses that encodes a homolog of the GTPase dynamin, and is expressed in many *C. elegans* cells[46].

The assembly and disassembly of peripheral actin filaments is an important requirement to facilitate changes in the structure of membranes during phagocytosis. Crucial regulators of actin-based cytoskeleton rearrangement as a consequence of apoptotic cell recognition include Rho GTPases (Rho,

Rac, and Cdc42) and phospatidylinositol 3-kinase (PI-3-kinase) that play a role in the extension of pseudopodia and in the formation and maturation of the phagosome[47]. In addition, 12/15 lipoxygenase (LO) seems to be involved in actin polymerization during phagocytosis, being translocated from the cytosol to the plasma membrane, close to regions of contact with apoptotic cells[48].

### Phagocytosis of Apoptotic Cells and Disease

The modulation of phagocytic capacity for apoptotic cell clearance represents a potential therapeutic target in the control of inflammatory disease since defects in clearance of apoptotic cells have been closely associated with several chronic inflammatory conditions, including systemic lupus erythematosus (SLE), in which autoantibodies against a number of self-antigens derived from apoptotic cells are developed[49,50]. Further evidence for the importance of efficient mechanisms for apoptotic cell clearance *in vivo* is supported by the observation that autoimmune responses can be provoked in mice when key molecules for apoptotic cell recognition and uptake, such as complement protein C1q, the tyrosine kinase receptor gene *Mer*, and MFG-E8, are missing or mutated[51,52,53]. Interestingly, an increased number of apoptotic cells has been demonstrated in the airways of patients with cystic fibrosis (CF) and non-CF bronchiectasis, probably as a consequence of elastase-mediated cleavage of the PS receptor on phagocytes[54].

## LIPOXINS: ENDOGENOUS MEDIATORS OF THE RESOLUTION OF INFLAMMATION

### Biosynthesis of Lipoxins

As already discussed, resolution of inflammation is related to a balance and temporal switch of proinflammatory and endogenous anti-inflammatory signaling systems. Among these signals, LXs have emerged as mediators of key events in endogenous anti-inflammation and proresolution[3,4,5,6,7,8]. They are typically formed by transcellular metabolism (Fig. 2) initiated by sequential oxygenation of arachidonic acid by both 5- and 12-LO or 15- and 5-LO[55,56,57,58]. In a cytokine-primed milieu, aspirin acetylation of cyclooxygenase-2 (COX-2) switches the catalytic activity of the enzyme to an *R*-LO with the formation 15R-HETE that is rapidly converted by 5-LO of activated PMN to 15-epimeric-LX (ATLs) (Fig. 2) The ATLs share many of the biological actions of the native LXs, albeit with greater potency and efficacy[55,56,57,58]. Although LXs and ATLs are generated by different routes, they act at the same receptor and evoke similar anti-inflammatory and proresolution actions[55,56,57,58].

LXs act locally and are rapidly inactivated by dehydrogenation at C15 and possibly by ω-oxidation at C20[55,57,58]. The 15-epi-$LXA_4$ is less readily converted to 15-oxo derivative than native $LXA_4$, indicating that the dehydrogenation step is stereospecific[59]. Biologically stable analogues of LX and ATLs were designed with specific modifications of the native structures of LXs[60,61,62]. The availability of LX analogues active *via* oral, topical, and systemic routes will facilitate studies on the functions and therapeutic applications of LX *in vivo*.

### Formation of Lipoxins and Aspirin-Triggered Lipoxins *In Vivo*

$LXA_4$ is generated *in vivo* within an inflammatory milieu. It may be suggested that impaired LX biosynthesis may correlate with an inability to resolve the acute inflammatory reaction contributing to a more chronic inflammatory phenotype. $LXA_4$ formation has been demonstrated in an immune complex model of glomerulonephritis[63], in pleural exudates on allergen challenge in rats[64], and in ischemic lungs[65]. There is a growing body of evidence that indicates an immunomodulatory role for LX during infections. $LXA_4$ levels generated during microbial infection with *Toxoplasma gondii* in a murine model are remarkably increased during the acute phase and stay high during chronic disease, probably a mechanism to suppress host defense[66,67]. It is possible to speculate that pathogens might take advantage of this regulatory pathway to promote host survival or even to allow a less-toxic environment in which replication can occur.

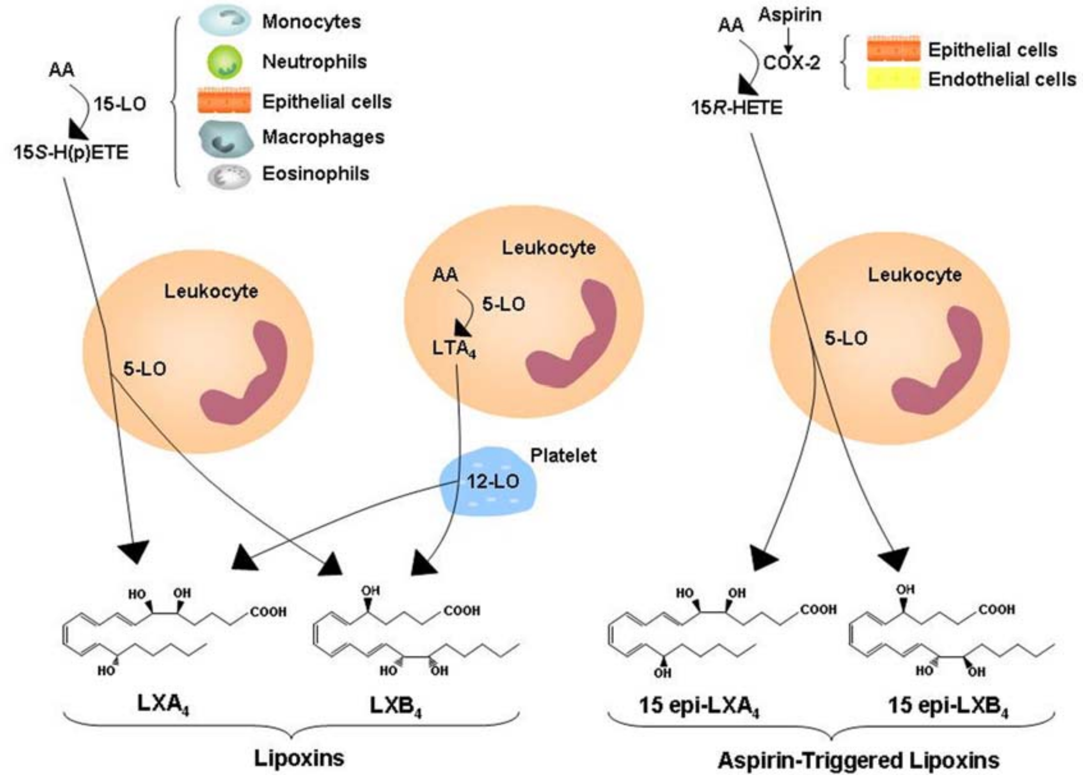

**FIGURE 2.** Biosynthesis of LXs and ATLs. During cell-cell interaction, LXs can be generated by transcellular biosynthesis via interaction of two or more cells. Two main classic LX-generating pathways involve the interaction of 5- and 12-LO or 5- and 15-LO. A third pathway involves the up-regulation of COX-2 and acetylation by aspirin. This acetylated COX-2 switches the enzyme activity with the production of 15R-HETE , the precursor of 15-epi-LXs (ATLs).

A reduction in LX production has been demonstrated in human diseases, such as airway inflammation[68], CF[69], and glomerulonephritis[70]. In contrast, $LXA_4$ production is up-regulated in localized juvenile periodontitis, limiting further PMN recruitment and PMN-mediated tissue injury[71], mild asthma[72], and in tissue-derived samples from nasal polyps[73]. The observation that $LXA_4$ was rapidly generated *in vivo* in atherosclerotic plaque rupture after angioplasty was the first demonstration that there is much greater abundance of proinflammatory mediators, such as LTs relative to the amounts of LXs, during chronic inflammatory disease[74]. The ratio of LT and LX formation at the site of inflammation *in vivo* might be relevant to understand the outcome of local inflammation. A reduction in LX formation may be deleterious leading to a chronic, local inflammation.

Recently, using a newly developed specific ELISA method, it was possible to detect 15-epi-$LXA_4$ *in vivo*, such as in an aspirin-dependent manner in murine peritonitis[75], dorsal air-pouches[76], and in rat kidney[63]. ATLs are formed in rat stomach after aspirin administration indicating that ATLs production is one of the mechanisms of gastric adaptation to aspirin[77]. Aspirin-intolerant asthmatics display lower biosynthetic capacity of LX and 15-epi-LX associated to a lower ratios for $LXA_4/LTC_4$ generation compared to aspirin-tolerant asthmatics[78]. Administration of low doses of aspirin to healthy subjects was shown to increase plasma levels of ATLs significantly with a concomitant inhibition of thromboxane biosynthesis[79]. Interestingly, the amounts of ATLs generated are sufficient to evoke and account for the anti-inflammatory action of ATLs[79]. Thus, ATLs formation *in vivo* provides a novel mechanism underlying the clinical benefits of aspirin, triggering anti-inflammatory mediator production that, in turn, dampen inflammation.

## Lipoxin Receptor

$LXA_4$ and ATLs bind to at least one G protein-coupled receptor that has been cloned, characterized, and designated as ALXR[55,56,57,58,80,81]. ALXR, belonging to the cluster of chemoattractant peptide receptors, is expressed in neutrophils[82], monocytes[83], activated T cells[84], basolateral membrane of gastrointestinal epithelial cells[85], synovial fibroblasts[86], bronchial epithelial cells[87], and mesangial cells[88]. ALXR was originally identified as a low-affinity N-formyl-methionyl-leucyl-phenylalanine (fMLP) receptor-like 1 (FPRL1) and there is considerable evidence that ALXR can bind pleiotropic ligands, i.e., both lipid and peptides (Table 1 and extensively reviewed in [80,81]). The binding of lipids and small peptides to the receptor occurs with different affinities and/or distinct interaction sites, facilitating activation of distinct signaling pathways that depend on the cell type and system[89].

**TABLE 1**
**Ligands for Lipoxin Receptor**

| |
|---|
| $LXA_4$, ATLs, and ATLs analogues |
| Glucocorticoid-inducible protein annexin-1 |
| MHC binding peptide (a potent necrotactic peptide derived from NADH dehydrogenase subunit 1 from mitochondria) |
| MMK-1 peptide |
| Antimicrobial peptides (e.g., LL37 and temporin A) |
| Truncated chemotactic peptides (e.g., CKbeta8-1) |
| A urokinase-type plasminogen activator receptor (uPar) fragment |
| HIV envelope peptides |
| Prion protein |
| Serum amyloid A |
| Amyloid $\beta_{42}$ |
| Glucocorticoid-inducible protein annexin-1 and peptide Ac2-26 Humanin |

For original references, see [55,56,57,58,80,81].

## Bioactions of Lipoxins

LXs have been shown to modulate specific actions in cells of both myeloid and nonmyeloid origin typically consistent with the distribution of the ALXR (for an extensive list of bioactions of LXs and ATLs see reviews[55,57,58]). LXs, ATLs, and stable synthetic LX analogues inhibit PMN and eosinophil chemotaxis[90,91], as well as PMN adhesion to and transmigration across endothelial cells and intestinal epithelia[92,93]. Both LXs and ATLs antagonize many of the effects of proinflammatory LTs including PMN-endothelial cell adhesion mediated by CD11/CD18 expression[92], endothelial PMN adhesion dependent on endothelial P-selectin[94], and integrin clustering and mobility on PMN[95]. LXs actively promote resolution of inflammation retarding the entry of new PMNs to sites of inflammation and reperfusion injury[65]. They also reduce vascular permeability[96], promote the nonphlogistic infiltration of monocytes that seems to be required for wound healing[97], and stimulate phagocytosis of apoptotic cells (see below).

LXs and ATLs have been shown to play a key role in regulating cytokine-chemokine axes directly modulating the cytokine composition in the inflammatory environment. In activated human synovial fibroblasts, LXs inhibit the synthesis of inflammatory cytokines and matrix metalloproteinases while stimulating tissue inhibitor of metalloproteinase expression[86]. LXs and LX analogues inhibit IL-8 from TNF-α-primed colonic cell lines[98], human colon *ex vivo*[99], and intestinal epithelia in response to challenge with *Salmonella typhimurium*[100]. Interestingly, ALXR is preferentially expressed on the basolateral surface of intestinal epithelia; therefore, LX generation at the paracellular space via neutrophil-epithelial interactions can rapidly act on the receptor to down-regulate intestinal inflammation[85].

## Lipoxins and Phagocytosis of Apoptotic Cells

As previously discussed, native LXs and ATLs are well-described “braking signals” in inflammation[55,56,57,58]. In contrast to inhibiting PMN function, LXs are potent activators of monocytes, stimulating their chemotaxis and adherence without causing degranulation or release of reactive species[97]. This observation suggested that LXs might be involved in the recruitment of monocytes to sites of wound healing or clearance. We have shown that native LX, ATLs, and stable synthetic LX analogues promote the resolution of inflammation, stimulating nonphlogistic phagocytosis of apoptotic PMNs and lymphocytes by Mϕ *in vitro* and *in vivo* in a murine model of thioglycollate-induced peritonitis[101,102,103]. LX-stimulated phagocytosis is associated with increased TGF-β1 release from Mϕ and a suppression of IL-8 and monocyte chemoattractant protein-1 release, consistent with a role for LX in promoting the resolution of inflammation[101,102]. The effect of LXs on phagocytosis of apoptotic PMNs by Mϕ can be blocked by antibodies to several macrophage surface proteins known to contribute to the recognition of apoptotic leukocytes, such as CD36, $\alpha_v\beta_3$, and CD11b/CD18, and it is mediated by protein kinase C and PI-3-kinase[101,102]. A modulatory role for cAMP is suggested by the observation that LX-induced phagocytosis is inhibited by a cell permeant cAMP analogue and mimicked by a protein kinase a (PKA) inhibitor[101]. Furthermore, LXs might prime Mϕ for chemotaxis and phagocytosis, inducing changes in the ultrastructure and reorganization of actin in human monocytes and Mϕ, resulting in the promotion of cytoplasmic extensions and in the formation of pseudopodia (Figs. 3 and 4) with a mechanism that is dependent on activation of the GTPases RhoA and Rac[104]. In addition, $LXA_4$-stimulated phagocytosis is associated with decreased phosphorylation and redistribution of MYH9, a nonmuscle myosin H chain II isoform A, involved in cytoskeleton rearrangement and polarization of Mϕ with activated Cdc42 localized toward the leading edge and MYH9 at the cell posterior[103]. $LXA_4$ stimulates the phosphorylation of polarity organization molecules, such as Akt, protein kinase Cζ, and glycogen synthase kinase-3β, suggesting that the effect of $LXA_4$ on cell polarization is a key early event in LX-induced phagocytosis[103].

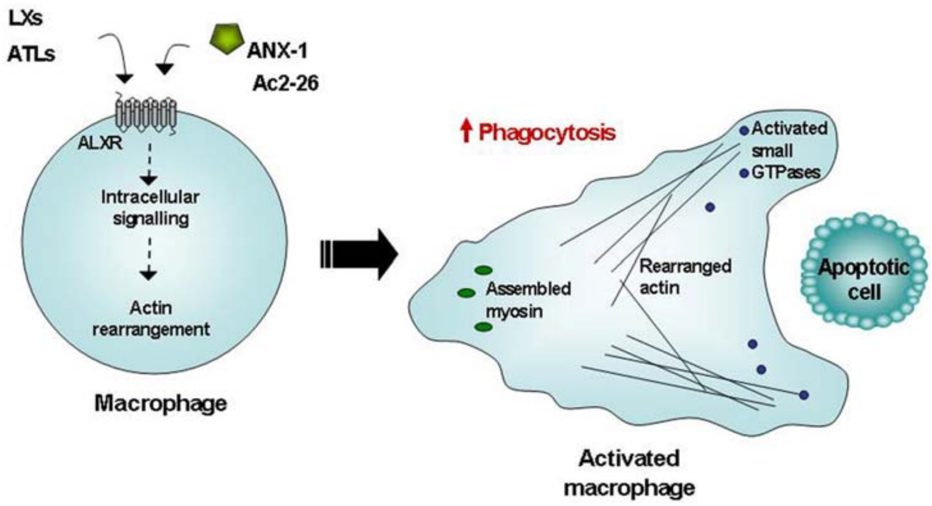

**FIGURE 3.** Phagocytosis of apoptotic cells by Mϕ is augmented by ligands of the LX receptor (ALXR). LXs and ATLs, annexin-1 (ANX-1), and its N-terminal fragment Ac2-26, interact with the ALXR on the macrophage leading to intracellular signaling events, including activation of the small GTPases RhoA, Rac, and Cdc42, myosin assembly and actin rearrangement, priming the Mϕ for the phagocytosis of apoptotic cells.

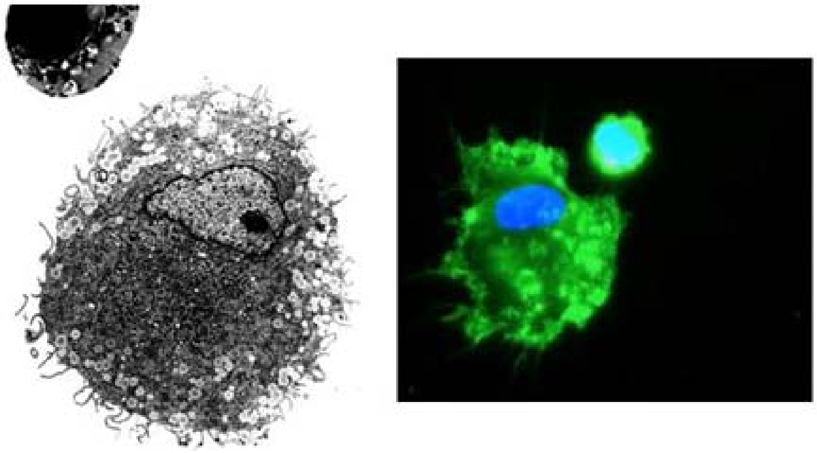

**FIGURE 4.** $LXA_4$ primes human monocyte-derived Mϕ to phagocytose apoptotic PMNs. Mϕ were treated with $LXA_4$ (1 nM for 15 min) before coincubation with aged PMNs for 30 min. Cells were fixed and processed for electron microscopy or immunofluorescence. Actin is visualized by staining with Oregon Green phalloidin.

Bone marrow–derived Mϕ (BMDM) are uncommitted phagocytes that can develop phenotypically distinct properties after cytokine programming. Interferon γ (IFNγ)/TNF-α–stimulated-BMDM are characterized by sustained nitric oxide (NO) production and a diminished phagocytic capacity, whereas exposure of BMDM to TNF-α stimulates phagocytosis of apoptotic PMN and is not associated with NO release, characteristic of a reparative phenotype. Intriguingly, exposure of TNF-α–programmed Mϕ to LX further enhances their ability to phagocytose apoptotic PMN, whereas LX rescues the compromised phagocytic activity of IFNγ/TNF-α–primed BMDM[102].

The beneficial effect of LXs on phagocytosis of apoptotic cells may contribute to expand the potential role of LXs in the treatment of diseases in which impaired apoptotic cell clearance has been demonstrated, i.e., CF[69]. The dysregulated proinflammatory environment of the CF airway, characterized by up-regulated IL-8 production and persistent, destructive neutrophilic inflammation, is consistent with a deficiency in LX-mediated anti-inflammatory activity[69]. In addition, the ability of ALXR to interact with amyloids[105] suggests that it may have a role in modulating the accumulation of extracellular amyloid, a key feature of neurodegenerative disorders.

## ANNEXIN-1

Annexins (also called lipocortins) are a family of structurally related, calcium-dependent, phospholipid binding proteins, defined by a highly conserved protein core domain (~70 amino acids) containing $Ca^{2+}$ and phospholipid binding sites, and an N-terminal region that is unique to each member of the family[106]. Annexin-1, one of the more studied members of the family, was originally identified in leukocytes as a glucocorticoid-inducible protein and termed lipocortin[107]. Annexin-1 was shown to mimic the anti-inflammatory effects of glucocorticoids in several experimental models of inflammation both *in vivo* and *in vitro*[108]. Passive immunization studies[109] and the development of annexin-1 null mice[110] have allowed a better definition of the roles played by the endogenous protein, principally in the inflammatory milieu in several cellular functions including phagocytosis, extravasation, and mediator generation. Annexin-1 expression is particularly abundant in cells of the innate immune system including monocytes and neutrophils[111,112], as well as Mϕ and epithelial cells[113], and it is secreted by the human prostate gland[114]. It has been demonstrated that neutrophil adhesion to endothelial monolayers *in vitro* mobilized large amounts of the protein on the leukocyte cell surface, probably through a process of controlled exocytosis[115]. More recently, Arur et al. have shown that during apoptosis, annexin-1 is recruited from the cytosol of Jurkat T lymphocytes and exported to the outer cell membrane where it colocalizes with PS[25].

## Bioactions of Annexin-1

Annexin-1 and annexin-1 mimetics (shorter peptide sequences from the N-terminal region of the protein), such as peptide Ac2-26, have anti-inflammatory effects in several models of acute and chronic inflammation[116,117,118,119,120]. Annexin-1 was originally described as an inhibitor of lipid mediator formation via blockade of phospholipase $A_2$ activity, thereby modulating many biological processes including cellular growth and differentiation, central nervous system response to cytokines, neuroendocrine secretion, and tissue neutrophil accumulation[116,117,118,119,120]. Studies have shown that annexin-1 may affect other enzymes involved in inflammation, such as the inducible nitric oxide synthase (iNOS) expression in rats with septic shock[121]. Additionally, dexamethasone and a N-terminal fragment of annexin-1 (residues 1-188) inhibited induction of iNOS in a macrophage cell line activated by lipopolysaccharide (LPS), an effect abrogated by a specific polyclonal antibody against annexin-1[121]. Others have shown that dexamethasone and annexin-1 can inhibit expression of COX-2 and iNOS as well as the release of $PGE_2$[122]. It has been suggested that that these effects of annexin-1 may be attributable to release IL-10 and a concomitant reduction of IL-12[123].

The process of leukocyte extravasation is sensitive to annexin-1 as shown by the reduction in number of leukocytes that adhere to and migrate through the inflamed postcapillary venule endothelium following administration of annexin-1 or its peptides[116,117,118,119,120]. Recently, differential effects of the full-length protein and Ac2-26 has been shown in neutrophil-endothelium interactions under flow, demonstrating that annexin-1 inhibited firm adhesion of human neutrophils, while Ac2-26 significantly attenuated capture and rolling without effect on firm adhesion[124]. Adhesion of monocytes to endothelium has also been shown to be strongly inhibited by exogenous and endogenous annexin-1[125]. Moreover, Ac2-26 down-regulates the accumulation of monocytes in zymosan-induced peritonitis[126].

Annexin-1 mimics many of the anti-inflammatory actions of glucocorticoids in experimental models of inflammation[116,117,118,119,120]. It has been reported that Mϕ-derived inflammatory mediators can be inhibited by glucocorticoids in an annexin-1 dependent manner including TNF-α and $PGE_2$ release from peripheral blood mononuclear cells[127]. Like glucocorticoids, annexin-1 and derived peptides inhibit eicosanoid synthesis[128], block leukocyte migration[115], and induce apoptosis in inflammatory cells[129].

Ac2-26 can reproduce the anti-inflammatory effects of the full-length annexin-1 molecule in a number of processes including neutrophil recruitment, hyperalgesia, and tissue injury caused by artery occlusion and reperfusion shock[124]. A novel effect for Ac2-26 was recently described by Bandeira-Melo et al. who demonstrated that in a model of rat pleurisy, Ac2-26 inhibited mast cell degranulation, plasma protein leakage, and accumulation of both neutrophils and eosinophils[130].

Defects in annexin-1 function are implicated in several inflammatory diseases. Antibodies to annexin-1 may be responsible for some forms of glucocorticoid resistance in rheumatoid arthritis (RA) patients, associated with the pathology of SLE[131,132], and Crohn's disease[133]. In patients suffering from RA, discrete annexin-1 expression has been observed in monocytes of the synovial membrane[134]. Interestingly, in a rat model of adjuvant-induced arthritis, it was concluded that endogenous annexin-1 mediated the anti-inflammatory effect of dexamethasone through the modulation of synovial TNF-α release and neutrophil recruitment[135]. Annexin-1 is down-regulated in CF knock-out mice lacking the CF transmembrane regulator gene, and in CF patients, a decreased level of annexin-1 correlated with a more severe phenotype[136]. An earlier study by Coméra et al. showed that in an experimental model of rat colitis, annexin-1 was secreted by neutrophils located in the inflammatory site[137].

## Annexin-1: Ligand for Lipoxin Receptor

Perretti et al. demonstrated that neutrophil-derived annexin-1 could be selectively immunoprecipitated with ALXR/FPRL1 when the leukocytes were adhered to endothelial monolayers[138]. As previously discussed, ALXR belongs to the family of human receptors for bacteria-derived formyl peptides, which also includes formyl peptide receptor (FPR) and FPR-like 2 (FPRL2). The peptides derived from the N-terminal region of annexin-1 have also been reported to bind to FPR as determined by receptor internalization in transfected cells and by blocking their actions with putative FPR antagonists[138]. In particular, while peptide Ac2-26 binds both FPR and ALXR/FPRL1, the full-length annexin-1 binds ALXR/FPRL1 only[124].

Although annexin-1 and its peptide derivatives can compete with $LXA_4$ for binding to ALXR[138], such binding can lead to ligand-specific signal transduction. For example, annexin-1[139] and its N-terminal peptides[140] cause shedding of L-selectin following addition to neutrophils (and monocytes) *in vitro*, whereas $LXA_4$ increases basal cell surface levels[141]. The observations that LXs and different peptides may interact with ALXR with different affinities and/or distinct interaction sites, evoking different intracellular signaling, may represent the need for multirecognition and receptor redundancies in the immune system.

## Annexin-1 and Phagocytosis

A role for endogenous annexin-1 in phagocytosis of apoptotic cells has been supported by the observation that annexin-1 is exported from the cytosol to the plasma membrane of apoptotic Jurkat T lymphocytes in a caspase-dependent manner and colocalizes with PS in discrete patches[25]. The dose-dependent reduction in tethering of phagocytes to apoptotic targets in which annexin-1 was down-regulated could be recovered when purified, endogenous annexin-1 was added[25]. Furthermore, the down-regulation of *C. elegans* annexin homolog nex-1 prevented efficient engulfment of cells corpses[25]. Fan et al. have reported that phagocytosis of apoptotic lymphocytes by Mϕ was inhibited by pretreatment of either target cells or phagocytes with an antibody to annexin-1, suggesting a bridging role for annexin between phagocyte and apoptotic target[142]. Using annexin-$1^{-/-}$ mice, Yona et al. demonstrated that when annexin-$1^{-/-}$ peritoneal Mϕ were incubated with opsonized zymosan particles or IgG complexes, they exhibited no difference in rate or maximal response in phagocytosis compared to wild-type mice[143]. However, when incubated with nonopsonized zymosan particles, the annexin-$1^{-/-}$ peritoneal Mϕ exhibited impaired generation of reactive oxygen species, and reduced phagocytosis of nonopsonized zymosan particles[143]. Additionally, bone marrow–derived annexin-$1^{-/-}$ Mϕ displayed a reduced phagocytic capacity for zymosan particles *in vitro* and *in vivo,* and an exacerbated release of cytokines following addition of inflammatory stimuli[144]. The ablation of the annexin-1 gene did not cause any apparent cytoskeletal defects associated with particle ingestion, but the cell surface expression of the key adhesion molecule CD11b was depressed in the annexin-$1^{-/-}$ cells, providing a possible explanation for the attenuated rate of phagocytosis[144].

A role for annexin-1 in the phagocytosis of apoptotic cells is further supported by our finding that annexin-1, released by dexamethasone-treated Mϕ, and the annexin-derived peptide Ac2-26, modulate macrophage phagocytosis of apoptotic neutrophils through a mechanism involving ALXR[145]. This effect is coupled to TGF-$\beta_1$ release and to changes in F-actin reorganization in Mϕ and MYH9 dephosphorylation and redistribution[103,145]. These data support the hypothesis that the Mϕ-derived annexin-1 could be a mediator of the described effects of glucocorticoids to enhance phagocytosis of apoptotic leukocytes[146,147]. We also showed that bone marrow–derived annexin-$1^{-/-}$ Mϕ displayed a greatly reduced phagocytic capacity to phagocytose apoptotic neutrophils when compared to wild-type mice[145]. Annexin-1, and particularly those peptides derived from its N-terminal region, might be attractive therapeutic targets in the treatment of inflammatory conditions.

## CONCLUSIONS

The uptake of apoptotic cells by professional phagocytes such as Mϕ is an important step in the resolution of inflammation that may be harnessed for therapeutic gain. However, more studies are necessary to define a clear link between impaired phagocytosis of apoptotic cells and inflammatory diseases. The potential proresolution activities of LXs and LX receptor agonists may be of benefit in this context. The observation that $LXA_4$ and annexin-1 act as ligands for ALXR might help in understanding the resolution phase of inflammation and expand the targets to develop new therapeutical approaches. Additionally, the growing appreciation of the role of nonprofessional phagocytes in disposal of apoptotic cells, coupled to the diversity of cell types responsive to LXs, suggest that this may be a more widespread phenomenon than previously thought. The powerful anti-inflammatory and proresolution actions of endogenous and aspirin-triggered LXs, coupled to their efficacy *in vivo*, suggest these agents possess therapeutic potential for use in human disease.

## ACKNOWLEDGMENTS

We would like to thank David C. Cottell from The Electron Microscopy Laboratory, Core Technology, UCD Conway Institute, for the transmission electron micrograph. We thank Prof. Catherine Godson for continued support and helpful discussions. Work in the authors' laboratory is supported by The Health Research Board Ireland, The Wellcome Trust, The Irish Research Council for Science Engineering and Technology, the European Union FP6 Grant EICOSANOX (LSHM-CT-2004-005033), and funded under the Programme for Research in Third Level Institutions by the Higher Education Authority.

## ABBREVIATIONS USED IN THE TEXT

| | |
|---|---|
| $\alpha_v\beta_3$ = | vitronectin receptor |
| 15R-HETE = | 15*R*-hydroxyl-5,8,11-*cis*-13-*trans*-eicosatetraenoic acid |
| ALXR = | lipoxin A4 receptor |
| ATL= | aspirin triggered lipoxin, 15-epi-$LXA_4$ (5*S*, 6*R*, 15*R*-trihydroxyl-7,9,13-*trans*-11-*cis*-eicosatetraenoic acid) |
| BMDM = | Bone marrow derived-macrophages |
| CF = | cystic fibrosis |
| COX-2 = | cyclooxygenase-2 |
| fMLP = | N-formyl-methionyl-leucyl-phenylalanine |
| FPR = | formyl peptide receptor |
| FPRL1 = | formyl peptide receptor-like 1 |
| Gas-6 = | growth arrest specific gene 6 |
| IFN$\gamma$ = | Interferon $\gamma$ |
| IL = | interleukin |
| iNOS = | inducible nitric oxide synthase |
| LO = | lipoxygenase |
| LPS = | lipopolysaccharide |
| LT = | leukotriene |
| $LXA_4$ = | lipoxin $A_4$ (5*S,* 6*R*, 15*S*-trihydroxyl-7,9,13-*trans*-11-*cis*-eicosatetraenoic acid) |
| $LXB_4$ = | lipoxin $B_4$ (5*S*, 14*R*, 15*S*-trihydroxyl-7,9,13-*trans*-11-*cis*-eicosatetraenoic) |
| M$\phi$ = | macrophages |
| MFG-E8 = | milk-fat globule epidermal growth factor 8 |
| NO = | nitric oxide |
| PG = | prostaglandin |
| PI-3-kinase = | phospatidylinositol 3-kinase |
| PKA = | protein kinase A |
| PMN = | polymorphonuclear neutrophils |
| PS= | phosphatidylserine |
| RA = | rheumatoid arthritis |
| SLE = | systemic lupus erythematosus |
| TGF-$\beta$1 = | transforming growth factor-$\beta$1 |
| TNF-$\alpha$ = | tumour necrosis factor-$\alpha$ |
